# A Specific Collagen Hydrolysate Improves Postprandial Glucose Tolerance in Normoglycemic and Prediabetic Mice and in a First Proof of Concept Study in Healthy, Normoglycemic and Prediabetic Humans

**DOI:** 10.1002/fsn3.4538

**Published:** 2024-10-20

**Authors:** Estelle Grasset, François Briand, Nicolina Virgilio, Christiane Schön, Manfred Wilhelm, Benoit Cudennec, Rozenn Ravallec, Hairati Aboubacar, Sara Vleminckx, Janne Prawitt, Thierry Sulpice, Elien Gevaert

**Affiliations:** ^1^ Physiogenex SAS Escalquens France; ^2^ Rousselot BV Gent Belgium; ^3^ BioTeSys GmbH Esslingen Germany; ^4^ Department of Mathematics, Natural and Economic Sciences Ulm University of Applied Sciences Ulm Germany; ^5^ UMRT BioEcoAgro 1158 Univ. Lille Lille France

**Keywords:** peptides, postprandial glucose tolerance, specific collagen hydrolysate

## Abstract

In response to nutrients, intestinal L‐ and K‐cells naturally secrete glucagon‐like peptide 1 (GLP‐1). GLP‐1 regulates postprandial blood glucose by increasing insulin secretion, slowing down gastric emptying and inducing satiety. A selection of specifically developed collagen hydrolysates was screened for their ability to enhance natural GLP‐1 production in vitro. The best performing hydrolysate, H80 (Nextida GC), was orally administered at different doses to lean, normoglycemic mice and overweight, prediabetic mice. Lean mice were acutely challenged 45 min before an oral glucose load. While daily supplemented for 6 weeks, prediabetic mice were acutely challenged at day 21 and 34. Oral glucose tolerance, plasma insulin and GLP‐1 levels were assessed, and a gastric emptying assay performed in prediabetic mice. H80 significantly lowered the blood glucose response in lean and prediabetic mice, at a 4 g/kg dose (−25% and −36%, respectively), compared to vehicle. In chronically supplemented, prediabetic mice, acute H80 administration slowed down gastric emptying (−60%) after 21 days and increased plasma insulin (+166%) after 35 days of supplementation. H80 increased plasma active GLP‐1 in lean (+217%) and prediabetic (+860%) mice. Overall, the data indicate that the specific collagen hydrolysate, H80, has significant GLP‐1‐mediated effects on oral glucose tolerance in lean and prediabetic mice. Furthermore, effects on postprandial glucose tolerance were evaluated in a small, human, proof of concept study. H80 reduced the postprandial glucose response at a 5 g dose in healthy, normoglycemic and prediabetic participants. Oral supplementation with H80 might thus be a promising strategy to maintain normal glucose tolerance.

## Graphical abstract

Nutritional intervention using a specific collagen‐based supplement, demonstrates a promising strategy to reduce postprandial glucose tolerance in both lean, normoglycemic and overweight, prediabetic mice by increasing plasma active GLP‐1 levels, insulin secretion, and lowering gastric emptying. A first proof of concept study showed its potential to reduce the postprandial glucose response in healthy, both normoglycemic and prediabetic, human participants.

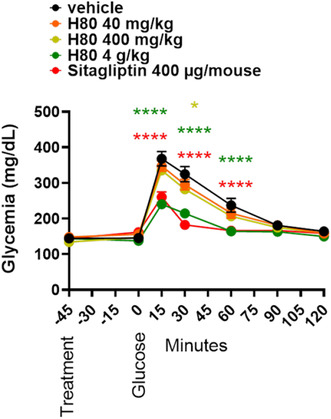

## 1 Introduction

Regulation and maintenance of blood glucose levels, at fasting and postprandially after a meal, are critical. High variability of blood glucose within the day can affect well‐being by causing depression, cravings, energy dips and an unhealthy eating pattern potentially leading to an increased risk of metabolic and cardiovascular disease in healthy individuals (Belli et al. 2023; Jarvis et al. 2023). In addition, dysregulated glucose tolerance in combination with overweight or obesity is a risk factor for the development of prediabetes and in later stages, type 2 diabetes (Weiss 2007). As a consequence, controlling glycemia is key to maintaining overall and metabolic health.

Glycemia is one of the most tightly regulated physiological parameters. Upon nutrient intake, the pancreas secretes insulin, a critical player in regulating postprandial blood glucose. Insulin secretion depends on efficient glucose detection by the β‐cells or on neuronal and hormonal pathways. The hormones glucagon‐like peptide‐one (GLP‐1) and gastric inhibitory polypeptide (GIP) are secreted by the intestinal L‐ and K‐cells, respectively, in response to nutrients such as glucose, fructose, free fatty acids and peptides (Hjorne, Modvig, and Holst 2022; Seino, Fukushima, and Yabe 2010). GIP exclusively enhances glucose‐induced insulin secretion (Seino, Fukushima, and Yabe 2010), while GLP‐1 additionally slows down gastric emptying and inhibits food intake (Holst 2007). GLP‐1 has a very short half‐life in the systemic circulation of less than a minute (Deacon 2004; Deacon, Johnsen, and Holst 1995) due to its degradation by the dipeptidyl peptidase 4 (DPP‐4), which also inactivates GIP (Seino, Fukushima, and Yabe 2010). In a diabetes type 2 context, endogenous GIP stimulation alone has been shown insufficient to lower blood glucose while in another study, both gastric emptying and the secretion of GLP‐1 were identified as the most relevant determinants of postprandial glycemia (Xie et al. 2023; Wu et al. 2014). Altogether, this indicates that boosting endogenous GLP‐1 secretion in combination with slowing gastric emptying is an attractive approach to optimize postprandial glycemia.

Based on its central role as regulator of glucose metabolism through different mechanisms, GLP‐1 related targets have been in focus for drug development. Several DPP‐4‐resistant GLP‐1 receptor agonists have been developed, e.g., liraglutide, lixisenatide, dulaglutide or semaglutide to cite a few (Trujillo, Nuffer, and Smith 2021). Another GLP‐1 based therapy inhibits the action to DPP‐4, e.g. sitagliptin or vildagliptin (Gallwitz 2019). Despite a high efficacy, each GLP‐1 based therapy acts differently to control blood glucose recruiting different molecular pathways compared to native GLP‐1 (Madsbad 2016). Indeed, none of them can exactly mimic all native GLP‐1 actions. In addition, most are characterized by side effects, such as gastrointestinal discomfort and cancer risk (Smits et al. 2016; Suryadevara et al. 2022).

Thus, targeting the natural secretion of native GLP‐1 by food or dietary supplements represents a promising strategy (Kamruzzaman et al. 2021), especially the use of hydrolyzed proteins as they directly target the enteroendocrine system. Interestingly, studies have suggested that protein source and applied hydrolysis conditions play a role (Miguens‐Gomez et al. 2021). Dietary, hydrolyzed collagen, a known source of bioactive peptides, provides an attractive approach as hydrolyzed collagen has been shown to achieve glycemic control via different modes of action (Sasaoka et al. 2021). In the present study, we selected a specific collagen hydrolysate for its capacity to enhance natural GLP‐1 secretion in enteroendocrine cells. We investigated the impact of this product on glucose tolerance in lean, normoglycemic and overweight, prediabetic mice, followed by a first proof of concept study in humans.

## 2 Materials and Methods

### 2.1 Test Compounds

A series of prototypes (CH1‐CH17) of porcine origin and an average molecular weight between 1000 and 8000 Da were selected from a proprietary library of specific collagen peptides. The hydrolyzed collagens were derived from the enzymatic hydrolysis of gelatin (predominantly type I collagen). All products were provided by Rousselot (Rousselot, Gent, Belgium).

### 2.2 In Vitro Simulated Gastrointestinal Digestion

In order to mimic human gastrointestinal digestion, a library of different collagen hydrolysates was digested in vitro by using the INFOGEST digestion protocol (Brodkorb et al. 2019), adapted for proteins only (Atallah et al. 2020). In brief, hydrolysates previously solubilized in 8 mL of water were mixed with 8 mL of salivary fluid, pH 7.0. After 5 min at 37°C, 8 mL of gastric fluid containing pepsin (Sigma‐Aldrich, St Louis, MO, USA, P6887, 6500 U mL^−1^) was added. Reaction media at pH 3.0 were incubated for 2 h at 37°C. Finally, 16 mL of intestinal fluid containing pancreatin (Sigma‐Aldrich, St Louis, MO, USA, P1750, 45 U mL^−1^ trypsin activity) was added. Reaction media at pH 7.0 were incubated for 2 h at 37°C. Final intestinal fluid was collected, heated at 95°C for 5 min for enzyme inactivation, centrifuged at 13,400 ***g*** for 5 min, and the resulting supernatant was kept at −20°C until further utilization.

### 2.3 GLP‐1 Release by STC‐1 Enteroendocrine Cells

The secretagogue activity of the in vitro digested collagen hydrolysates was assessed using the methodology described in a previous study (Tenenbaum et al. 2023). Concisely, murine enteroendocrine STC‐1 cells (Sigma‐Aldrich, St Louis, MO, USA) were cultured in Dulbecco's Modified Eagle's Medium (DMEM) (PAN Biotech, Aidenbach, Germany), supplemented with 10% (v/v) Foetal Bovine Serum (FBS), 100 U mL^−1^ of penicillin and streptomycin, and 2 L‐glutamine at 2 mM. Cells were routinely maintained at 37°C in a 5% CO_2_‐modified atmosphere in 75 cm^2^ flasks (Sarstedt, Nümbrecht, Germany). To prepare the GLP‐1 release experiment, STC‐1 cells were seeded in 24‐well plates at a density of 20,000 cells per cm^2^. They were grown at 37°C in a modified atmosphere until reaching 80% confluence. On the day of the experiment, cells were washed in PBS and incubated with in vitro digested collagen hydrolysate diluted at 10 mg mL^−1^ in specific HEPES buffer (4.5 mM KCl, 1.2 mM CaCl_2_, 140 mM NaCl and 20 mM Hepes, pH 7.4) for 2 h. The well media were recovered and centrifuged (1200 rpm, 5 min). The supernatants were stored at −80°C until the GLP‐1 concentration was measured using the Glucagon‐Like Peptide‐1 (7–36) Amide EIA kit, EK‐028‐11CE (Phoenix Pharmaceuticals, Burlingame, USA). Cell viability (> 95%) was first checked using the Cell Counting Kit‐8 (CCK‐8, Tebu‐Bio, Le Perray en Yvelines, France).

### 2.4 Animals

All animal protocols were reviewed and approved by the local (Comité régional d'éthique de Midi‐Pyrénées) and national (Ministère de l'Enseignement Supérieur et de la Recherche) ethics committees (protocol number CEEA‐122‐2014‐15). C57BL6/J mice (Janvier Labs, France), male, 8‐week‐old at delivery, were housed in groups of four to five mice in enriched and ventilated mouse cages (Techniplast GM500, 500 cm^2^ surface, 12.7 cm height) in a room with 22°C ± 2°C, and 50% ± 10% relative humidity, and a 12‐h day/night cycle.

For the lean, normoglycemic mouse model, the experimental design is shown in Appendix S1. During all experiments, mice had free access to a standard diet (SAFE, reference U8400G10R, Augy, France) and tap water. After 5 days of acclimation, mice were randomized into 4 treatment groups (*n* = 10 mice/group) according to their body weight before undergoing an oral glucose tolerance test.

For the diet‐induced overweight, prediabetic mouse model, the experimental design is shown in Appendix S2. After a 5‐day acclimation period and during the experimental period, 50 mice had free access to a 60 kcal% high‐fat diet (HFD, reference D12492 from Research Diets via Brogaarden, Lynge, Denmark) and tap water. Body weight was monitored weekly until the end of the experimental phase. After 6 weeks of diet, mice were weighed, 6‐h fasted prior to blood collection from the tail tip to randomize animals into 4 homogenous groups (*n* = 10/group) based on HOMA‐IR and body weight. HOMA‐IR was calculated from fasting blood glucose measured from the tail tip using a glucometer and fasting plasma insulin values determined by ELISA (Eurobio, Montpellier, France reference 80‐INSMSU‐E10) as HOMA‐IR = (mM glucose × μU/mL insulin)/22.5. Ten high‐fat‐fed mice showing extreme values (maximum and/or minimum) of HOMA‐IR and body weight were excluded from the study. Mice were then daily supplemented orally for 6 weeks with either vehicle or H80 at 3 different doses (10 mL/kg gavage volume, at 1, 2 and 4 g/kg doses, dissolved in tap water).

### 2.5 Oral Glucose Tolerance Test and Gastric Emptying Assay

An oral glucose tolerance test (oGTT) was performed in lean, normoglycemic, 8‐week‐old, male C57BL6/J mice. Mice were first fasted for 6 h and treated p.o. acutely 45 min before the oral glucose load (Sigma‐Aldrich, reference 30,970, 2 g/kg of body weight, dissolved in tap water) with either vehicle or the best performing hydrolysate from the in vitro experiment, H80 (Nextida GC), at 3 different concentrations 40 mg/kg, 400 mg/kg and 4 g/kg (10 mL/kg) and the DPP‐4 inhibitor sitagliptin (Carbosynth, United‐Kingdom, reference BS164409, 400 μg/mouse, dissolved in tap water). The DPPIV‐inhibitor sitagliptin was used as a positive control because it highlights the endogenous GLP‐1 secretion. Blood glucose was measured at times −45, 0, 15, 30, 60, 90 and 120 min after glucose load. Blood (15 μL/EDTA) was also collected at time 45 min before (−45 min) and time 15 min after (+15 min) glucose load to assay plasma insulin by ELISA. The insulin/glycemia ratio at time 15 min was calculated. The capacity of incretins to stimulate insulin secretion is influenced by the glucose levels around the β‐cell. Since glucose itself induces insulin secretion, a high glucose concentration can “hide” the incretin effect. Calculating the insulin/glucose ratio 15 min after the oral glucose load allows to estimate this incretin effect. A ratio superior to the one in the control group only receiving glucose indicates an incretin effect. The ratio of insulin +15 min/insulin −45 min was calculated for each individual to normalize the insulin level after glucose stimulation with the fasting insulin. Areas under the curve (AUC) were calculated as follows: ((glycemia at t0 + glycemia at t15)/2*(15–0)) + (glycemia at t15 + glycemia at t30)/2*(30–15) + ((glycemia at t30 + glycemia at t60)/2*(60–30)) + ((glycemia at t60 + glycemia at t90)/2*(90–60)) + ((glycemia at t90 + glycemia at t120)/2*(120–90)).

In overweight, prediabetic HFD‐fed mice the oGTT was performed at day 20 and day 34 of supplementation as described above except for the dose of H80, which was administrated 45 min before oral glucose load at 1, 2 and 4 g/kg. To evaluate the effect of H80 on gastric emptying, the oGTT was combined with a gastric emptying assay at day 20 of supplementation: mice were gavaged with a glucose‐acetaminophen solution (glucose: 2 g/kg of body weight; acetaminophen: 150 mg/kg, Sigma Aldrich reference A7085; 10 mL/kg body weight gavage volume). Blood (EDTA) was collected at time 0, 15, 30 and 60 min after bolus administration (0, +15, +30 and + 60 min) to assay plasma acetaminophen (colorimetric assay, Cambridge life sciences, reference K8001).

### 2.6 Incretin Assays

Lean, normoglycemic, 8‐week‐old, male, C57BL6/J mice and overweight, prediabetic HFD‐fed mice were fasted for 6 h. All mice were treated p.o. acutely with sitagliptin (400 μg/mouse, dissolved in tap water) to avoid the degradation of GLP‐1 and GIP by DPP‐4. 30 min later, mice received vehicle, H80 at 3 different doses 40 mg/kg, 400 mg/kg and 4 g/kg. 15 or 30 min after H80 treatment, mice were anesthetized intravenously with a Ketamin (10 mg/kg, Centravet, Castelnaudary, France)/Xylasin (1 mg/kg, Centravet, Castelnaudary, France) solution and blood was sampled from the portal vein. To avoid GLP‐1 and GIP degradation inside the tube, a specific anticoagulant solution mixed with antiprotease (diprotin A (1 mM), aprotinin (0.2 μM) and EDTA (1 mM)) was added. Plasma was then isolated after centrifugation (10,000 ***g***, 4°C, 5 min), and snap‐frozen in liquid nitrogen before active GLP‐1 and GIP levels were measured by ELISA assays (active GLP‐17‐36, ALPCO, #43‐GP1HU‐E01, Salem, USA; active GIP1‐42, Crystal Chem, #81511, Elk Grove Village, USA).

### 2.7 Human Proof of Concept Study

A randomized double‐blind, placebo‐controlled, three‐way cross‐over, with a 14 ± 7 days wash‐out period between visits, the study was conducted in accordance with the guidelines for Good Clinical Practice (GCP) and in accordance with the Declaration of Helsinki at BioTeSys GmbH, Esslingen, Germany. The clinical study was advised and approved by the ethics committee of the Landesärztekammer Baden‐Württemberg (F‐2023‐041) and registered at ClinicalTrials.gov (NCT05887791). All volunteers signed an informed consent form prior to the start of the study. Twenty‐two participants were screened for eligibility, and after informed consent, finally, 16 participants were randomized into the study as indicated in the Consort flow chart (Appendix S3). One prediabetic individual dropped out after visit 2. According to protocol, the participant was replaced and allocated in the same sequence group. Inclusion and exclusion criteria are described in Appendix S4.

Healthy, normoglycemic (*n* = 7) and prediabetic (*n* = 9) individuals were recruited. Fasting glucose of the normoglycemic participants was 92.3 mg/dL (95% CI: 89.1–95.4) and in prediabetic participants 111.0 mg/dL (95% CI: 103.3–118.7). Correspondingly, HbA1c values were significantly higher in prediabetic participants with 5.79% (95% CI 5.57–6.01) in comparison to 5.20% (95% CI: 5.03–5.37) in the normoglycemic participants. Reported medical history and intake of chronic concomitant medication was not in conflict with study participation. Further descriptive parameters of the overall study population and subgroups are shown in Table S1.

All participants arrived in the study center after an overnight fast of at least 10 h and underwent a mixed meal test (MMT) standardized to 75 g carbohydrates, comparing the postprandial blood glucose response to the MMT after a single dose intake of either H80 in two different dosages (5 g or 10 g) or placebo. Placebo consisted of 1 g cherry flavor only (FOODAROM, Weyhe, Germany), dosings of 5 g H80 and 10 g H80 were each masked with 1 g of cherry flavor. All administered samples were matched in taste and appearance. A single dose intake of placebo, 5 g H80 or 10 g H80 dissolved in water was administered 30 min prior to the MMT, comprising 110 g white toast, 20 g butter and 43 g strawberry jam. Its caloric content was 533.12 kcal (fat 20.90 g/194.37 kcal/36.43%; carbohydrate 75.08 g/307.83 kcal/57.74%; protein 9.16 g/37.56 kcal/7.04%) and adding a maximum of 10 g protein (10 g H80) equivalent to 41 kcal increased the caloric content by 7.69%.

Glucose concentrations were assessed in venous blood drawn from a catheter at the time points: −30 and 0 min prior to and 30, 60, 90, 120, 150 and 180 min after the intake of the MMT. The primary efficacy endpoint was defined as the incremental AUC between time points 0 and 180 min (iAUC_0–180_). iAUC and the maximum increase of the glucose concentration from t0 min as baseline (Δ*C*_max_) were calculated from individual concentration‐time curves.

### 2.8 Statistics

in vitro: Data are shown as mean ± SD (*n* = 3). Means without a common letter are significantly different (*p* < 0.05) using one‐way ANOVA followed by Tukey's test for pairwise comparisons.

in vivo: Data are shown as mean ± SEM. Statistical analysis was performed on GraphPad Prism using a Two‐way ANOVA + Bonferroni's post‐test, a 1‐way ANOVA + Dunnett's post‐test or if a significant different variance was found, a Kruskal–Wallis with Dunn's post‐test. A *p* < 0.05 was considered significant.

Human study: iAUCs of blood glucose were analyzed using a linear mixed model of iAUC with treatment (3 levels), period (3 levels), sequence (3 levels), and baseline blood glucose level within study periods as fixed effects and participant as a random effect. Data are presented for the intention to treat population (ITT). Multiple paired comparisons of least squares mean of iAUC were performed in order to assess differences between the two active treatments and placebo. Results are presented as least square means of iAUC differences between treatments against the mixed meal with placebo intake with associated 95% confidence intervals (CI). In addition, the percentage change was calculated from mean values. Furthermore, pharmacokinetic endpoints were evaluated by the subgroup of normoglycemic and prediabetic participants. For the secondary endpoint Δ*C*_max_, the same approach was applied.

## 3 Results

### 3.1 In Vitro Screening of Collagen Hydrolysates

**FIGURE 1:**
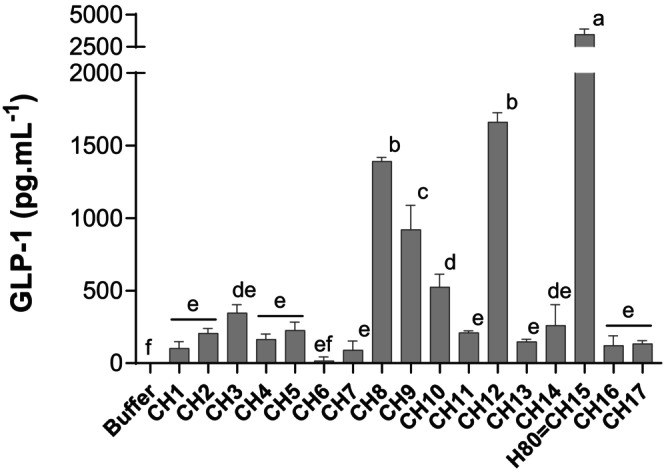
In vitro digested collagen hydrolysates differently stimulate GLP‐1 release. Seventeen in vitro digested collagen hydrolysates (CHx) were incubated for 2 h at 10 mg mL^−1^ (w/v) with STC‐1 enteroendocrine cells. Values are means ± SD (*n* = 3). Means without a common letter are significantly different (*p* < 0.05) using one‐way ANOVA followed by Tukey's test for pairwise comparisons.

To identify the most promising collagen peptide composition, 17 collagen hydrolysates (CHx) were generated by varying specific hydrolysis parameters and subsequently evaluated for their GLP‐1 stimulating capacity after in vitro gastrointestinal digestion. The digested hydrolysates were incubated for 2 h with STC‐1 enteroendocrine cells. All collagen hydrolysates (10 mg mL^−1^, *n* = 3) stimulated GLP‐1 secretion. The majority of digested hydrolysates (12) resulted in GLP‐1 concentrations recovered in cell supernatants ranging from 17 (CH6) to 346 pg mL^−1^ (CH3). Four of them yielded GLP‐1 levels ranging from 525 (CH10) to 1662 pg mL^−1^ (CH12). The digested hydrolysate exhibiting maximal GLP‐1 secretion, with a recovered concentration of 3475 pg mL^−1^, was CH15, representing a 33.4‐fold increase in secretion compared to the internal reference digested collagen hydrolysate (CH1) (Figure 1). CH15, hereafter referred to as H80, was therefore chosen as the most promising candidate for further testing in in vivo experiments.

### 3.2 In Vivo Validation in Lean, Normoglycemic Mice

**FIGURE 2:**
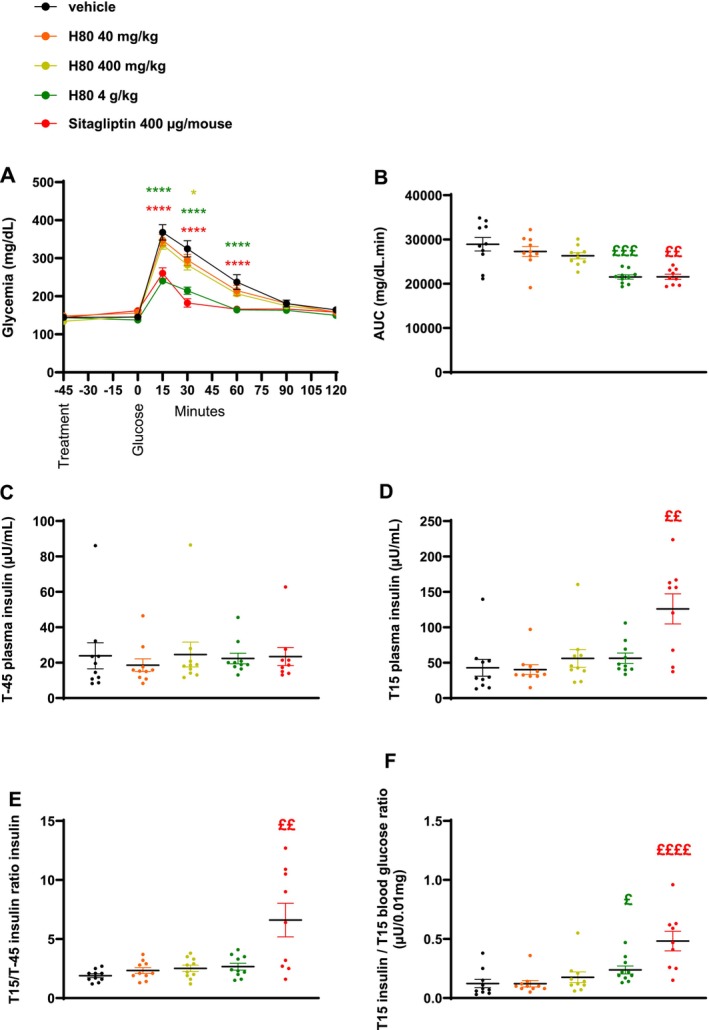
Effects of H80 on glycemia and insulin secretion during an oral glucose tolerance test in lean, normoglycemic mice. Mice were orally administered 45 min before glucose gavage: With vehicle, H80 at 40 mg/kg, 400 mg/kg or 4 g/kg or with sitagliptin (400 μg/mouse). (A) Glycemia, (B) glycemia AUC, (C) plasma insulin levels at fasting, just before H80 administration and 45 min before glucose administration (T‐45) and (D) 15 min after glucose gavage (T15). (E) Ratio between insulin levels 15 min after and 45 min before glucose gavage. (F) Ratio between insulin levels and glycemia 15 min after glucose gavage. Data are expressed as mean ± SEM, *n* = 9–10 per group. Statistical significance was evaluated for glycemia curves by two‐way ANOVA (followed by posthoc Bonferroni's multiple‐comparison tests) *p < 0.05, **p < 0.01, ***p < 0.001, ****p < 0.0001, and for the other parameters by one‐way ANOVA (followed by posthoc Dunnett's multiple‐comparison tests) *p < 0.05, **p < 0.01, ***p < 0.001, ****p < 0.0001 or if a significant difference of variance was observed by Kruskal–Wallis (followed by posthoc Dunn's multiple‐comparisons tests) £*p* < 0.05, ££*p* < 0.01, £££*p* < 0.001, ££££*p* < 0.0001.

To understand whether the strong GLP‐1‐inducing activity observed in vitro could be confirmed in an in vivo setting and could be linked to a physiological action, the acute effect of H80 on glucose tolerance was first evaluated in lean, normoglycemic mice during an oGTT. Compared to the vehicle control group, the H80 dose of 4 g/kg clearly and significantly decreased the glycemia between time points 15 and 60 min after the oral glucose load as effectively as the positive control sitagliptin (*p* < 0.0001 vs. vehicle, Figure 2A). While the two lower dosages of H80 showed a decreasing trend, no statistically significant effect on the glucose excursion was observed. Accordingly, the AUC of both the 4 g/kg H80 and the sitagliptin groups was reduced by 25% (H80: *p* < 0.001 and sitagliptin: *p* < 0.01 vs. vehicle, Figure 2B). At fasting, plasma insulin levels were similar in all groups (Figure 2C). Fifteen minutes after the oral glucose load, only the plasma insulin level of the sitagliptin‐treated group was increased compared to the vehicle control group (*p* < 0.01 vs. vehicle, Figure 2D). No effect of H80 was observed when plasma insulin levels at 15 min were divided by fasting insulin level at time −45 min while sitagliptin increased the ratio (*p* < 0.01 vs. vehicle, Figure 2E). The latter result confirmed the capacity of sitagliptin to induce insulin secretion in presence of glucose by inhibiting DPP4 and protecting GIP and GLP‐1 from degradation which clearly separates it from the functioning of H80. The capacity of incretins to stimulate insulin secretion is influenced by the glucose levels around the β‐cells. Calculating the ratio between insulin levels and the glycemia 15 min after the oral glucose load allows to estimate this incretin effect. The ratio was increased by H80 at the dose of 4 g/kg and sitagliptin (H80: +100%, *p* < 0.05 and sitagliptin: +327%, *p* < 0.0001 vs. vehicle Figure 2F). Overall, these data indicated that H80 at 4 g/kg reduces blood glucose levels through an incretin effect during an oGTT in lean, normoglycemic mice.

**FIGURE 3:**
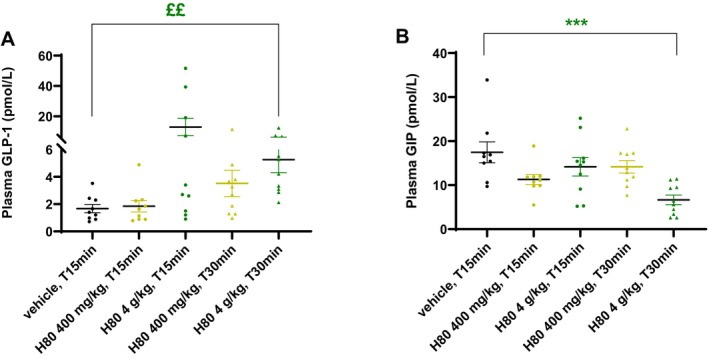
Effects of H80 on active GLP‐1 and GIP levels in lean, normoglycemic mice. Mice were orally administered with vehicle, H80 at 400 mg/kg or 4 g/kg and 15 min or 30 min after, active GLP‐1 (A) and GIP (B) levels were assessed in the portal vein. Data are mean ± SEM, *n* = 9–10 per group. Statistical significance was evaluated by one‐way ANOVA (followed by posthoc Dunnett's multiple‐comparison tests), ***p < 0.001, or if a significant different of variance was observed by Kruskal–Wallis (followed by posthoc Dunn's multiple‐comparison tests), ££*p* < 0.01.

To further confirm that H80 was able to increase plasma incretin levels in vivo as it was observed in vitro, fasted mice were orally administered with H80 15 and 30 min prior to blood sampling from the portal vein. H80 at 4 g/kg tended to increase plasma active GLP‐1 levels 15 min and significantly increased those levels 30 min after H80 dosing, when compared to vehicle (15 min: +673%, ns; 30 min: +217%, *p* < 0.01 vs. vehicle, Figure 3A). At the maximum dose, H80 reduced active GIP levels at time 30 min after H80 dosing (30 min: −61%, *p* < 0.001 vs. vehicle, Figure 3B). The effect on plasma GLP‐1 levels is in line with the observation on plasma GIP levels, since GLP‐1 inhibits GIP secretion (Pederson et al. 1998). The moderate effect of H80 on plasma insulin levels 15 min after the glucose load could be explained by the negative feedback of GLP‐1 on GIP secretion.

Taken together, the data collected in lean, normoglycemic C57BL6/J mice demonstrate that the highest dose of H80 was able to reduce glucose excursion after an oGTT and increase plasma GLP‐1 levels.

### 3.3 In Vivo Validation in Overweight, Prediabetic Mice

**FIGURE 4:**
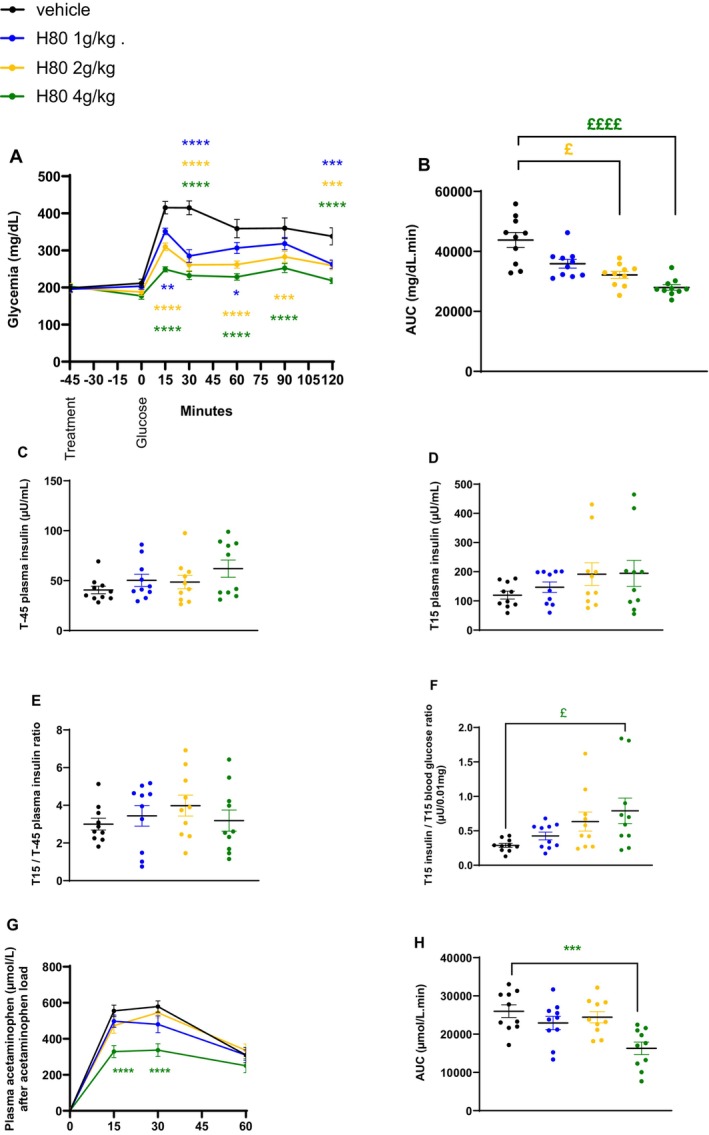
Effects of H80 on glycemia, insulin secretion and gastric emptying during an oral glucose tolerance test in overweight, prediabetic mice after 3 weeks of daily supplementation. Mice were orally supplemented daily for 3 weeks and orally administered 45 min before a glucose gavage with vehicle, H80 at 1, 2 or 4 g/kg. (A) Glycemia, (B) glycemia AUC, (C) plasma insulin levels 45 min before (T‐45) and (D) 15 min after glucose gavage (T15). (E) Ratio between insulin levels 15 min after and 45 min before glucose gavage. (F) Ratio between insulin levels and glycemia 15 min after glucose gavage. (G) Plasma acetaminophen levels and (H) AUC after acetaminophen gavage. Data are mean ± SEM, *n* = 10 per group. Statistical significance was evaluated for glycemia and acetaminophen curves by two‐way ANOVA (followed by posthoc Bonferroni's multiple‐comparison tests) *p < 0.05, **p < 0.01, ***p < 0.001, ****p < 0.0001, and for the other data by one‐way ANOVA (followed by posthoc Dunnett's multiple‐comparison tests) ***p < 0.001, ****p < 0.0001 or if a significant different of variance was observed by Kruskal–Wallis (followed by posthoc Dunn's multiple‐comparison tests) £*p* < 0.05, £££*p* < 0.001.

Since H80 showed beneficial effects on the glucose tolerance and plasma GLP‐1 levels in lean, normoglycemic mice, it was interesting to understand the effect of H80 in mice characterized by a dysregulation of glucose metabolism as in obese and prediabetic mice. Thus, mice were fed a high‐fat diet for 6 weeks (diet period) prior to a chronic, daily supplementation of H80 (3 different dosages) or vehicle in addition to the high‐fat diet for an additional 6 weeks (supplementation period). The mice gained approximately 10 g in the 6 weeks of the diet period (Figure S1A,B) and were randomized into 4 homogenous groups according to their HOMA‐IR and body weight (HOMA‐IR ~29 and body weight ~35 g, Figure S2A,B) before starting the supplementation. A HOMA‐IR around 29 characterizes mild insulin resistance and a body weight around 35 g at 15 weeks of age represents overweight in mice. As a reference, HOMA‐IR of lean, healthy mice with a body weight around 25–28 g is below 10 while HOMA‐IR of obese insulin‐resistant mice with a body weight around 45–50 g is between 50 and 100. During the 6‐week supplementation period, H80 had no effect on body weight except on the last day of supplementation, the lowest dose of H80 induced a significant increase of body weight change (H80 1 g/kg: +1.41 g vs. vehicle: −1.17 g, *p* < 0.05, Figure S3A,B). Indeed, after day 20 and 33 of treatment, body weight and body weight gain of mice in all groups decreased as a result of fasting followed by oGTT experiments. The significant difference between mice treated with vehicle or H80 at the lowest dose was probably the consequence of the vehicle group being less resistant to the impact of the previous fasting and oGTT procedure than the group treated with the lowest dose of H80. After 21 days of supplementation, an oGTT combined with a gastric emptying assay was performed. All tested doses of H80 significantly reduced glycemia between the time points 15 and 120 min (Figure 4A) compared to the vehicle control group resulting in a dose‐dependent reduction of the AUC (H80, 1 g/kg: −18%, ns; 2 g/kg: −26%, *p* < 0.05; 4 g/kg: −36%, *p* < 0.0001, vs. vehicle; Figure 4B). No effect of H80 was observed on plasma insulin levels at fasting, 15 min after the oral glucose load nor on the ratio between plasma insulin levels at 15 min after the oral glucose load and fasting (Figure 4C–E). The ratio between insulin levels and glycemia at 15 min after the oral glucose load was significantly increased in the group treated with H80 at the highest dose compared to the vehicle (*p* < 0.05, vs. vehicle; Figure 4F), indicating an incretin effect. Interestingly, plasma acetaminophen levels of the H80 4 g/kg supplemented group were significantly reduced at time points 15 and 30 min after the oral acetaminophen load (*p* < 0.0001; Figure 4G) leading to a significantly reduced AUC (−60%, *p* < 0.001, vs. vehicle, Figure 4H), indicating that H80 only at this highest dose delayed gastric emptying.

**FIGURE 5:**
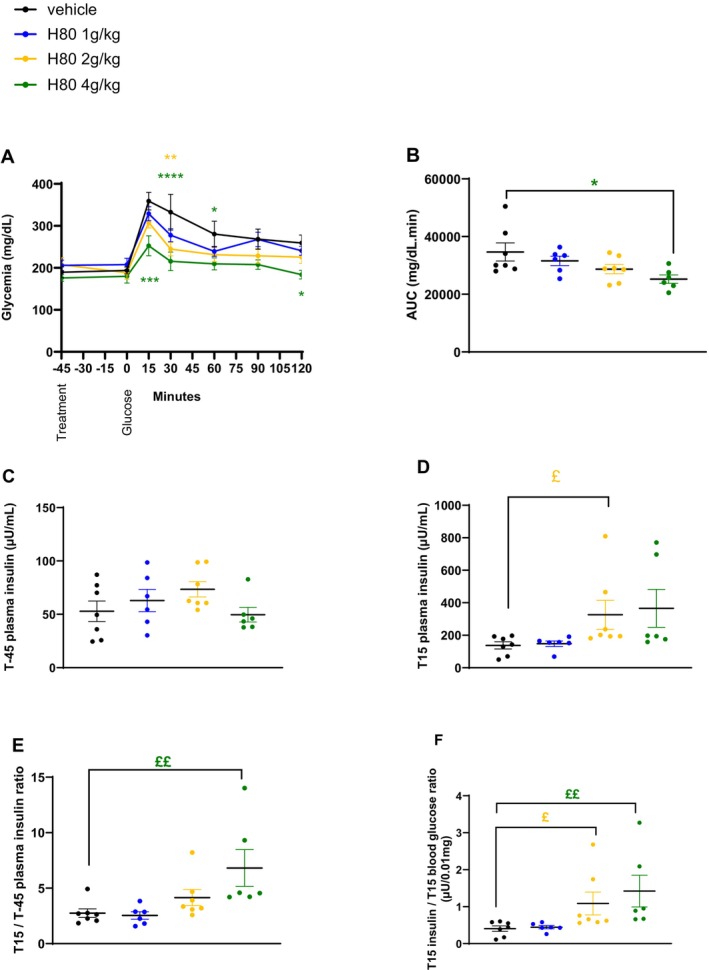
Effects of H80 on glycemia and insulin secretion during an oral glucose tolerance test in overweight, prediabetic mice after 5 weeks of daily supplementation. Mice were orally supplemented daily for 5 weeks and orally administered 45 min before a glucose gavage with vehicle, H80 at 1, 2 or 4 g/kg. (A) Glycemia, (B) AUC, (C) plasma insulin levels 45 min before (T‐45) and (D) 15 min after glucose gavage (T15). (E) Ratio between insulin levels 15 min after and 45 min before glucose gavage. (F) Ratio between insulin levels and glycemia 15 min after glucose gavage. Data are mean ± SEM, *n* = 6–7 per group. Statistical significance was evaluated for glycemia curve by two‐way ANOVA (followed by posthoc Bonferroni's multiple‐comparison tests) *p < 0.05, **p < 0.01, ***p < 0.001, ****p < 0.0001, and for the other data by one‐way ANOVA (followed by posthoc Dunnett's multiple‐comparison tests) *p < 0.05, **p < 0.01, ***p < 0.001, ****p < 0.0001 or if a significant difference of variance was observed by Kruskal–Wallis (followed by posthoc Dunn's multiple‐comparisons tests) £*p* < 0.05, ££*p* < 0.01.

**FIGURE 6:**
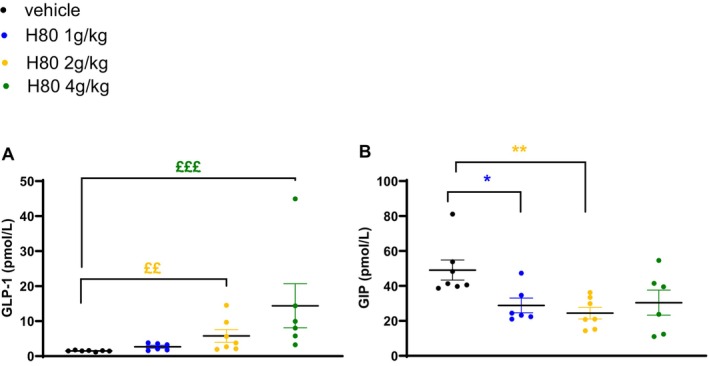
Effects of H80 on active GLP‐1 and GIP levels after 6 weeks of daily supplementation in overweight, prediabetic mice. Mice were orally supplemented daily for 6 weeks and orally administered 30 min before portal vein blood sampling vehicle, H80 at 1, 2 or 4 g/kg. Active GLP‐1 (A) and GIP (B) levels were assessed in the portal vein. Data are mean ± SEM, *n* = 6–7 per group. Statistical significance was evaluated by one‐way ANOVA (followed by posthoc Dunnett's multiple‐comparison tests) *p < 0.05, **p < 0.01, or if a significant difference of variance was observed by Kruskal–Wallis (followed by posthoc Dunn's multiple‐comparison tests), ££*p* < 0.01, £££*p* < 0.001.

Another oGTT was performed at the end of the supplementation period, i.e. after 35 days of supplementation. Only the highest dose decreased glycemia between time points 15–120 min (Figure 5A) resulting in a significant reduction of the AUC (−36%, *p* < 0.05, vs. vehicle Figure 5B). While no effect of H80 was observed on plasma insulin levels at fasting (Figure 5C), an increase of plasma insulin at 15 min after the oral glucose load was observed in the group treated with the two highest doses of H80 (2 g/kg: +138%, *p* < 0.05, 4 g/kg: +166%, *p =* 0.097, vs. vehicle, Figure 5D) leading to a significant increase of the ratio between plasma insulin levels at 15 min after the oral glucose load and at fasting (2 g/kg: +151%, ns, 4 g/kg: +248%, *p* < 0.01, vs. vehicle, Figure 5E). This result indicates that the chronic supplementation with H80 had a stronger effect on glucose‐induced insulin secretion at the end of the supplementation period (2 g/kg: 3.98 at week 3 vs. 4.15 at week 5; 4 g/kg: 3.18 at week 3 vs. 6.81 at week 5). The ratio between insulin level and glycemia at 15 min after the oral glucose load was significantly increased in the groups supplemented with H80 at the 2 highest doses (2 g/kg: +166%, *p* < 0.05; 4 g/kg: +246%, *p* < 0.01, vs. vehicle; Figure 5F), indicating a stronger incretin effect at the end of the supplementation period. This latter result was confirmed by measuring plasma incretin levels in the portal vein. H80 at all doses increased plasma GLP‐1 levels (1 g/kg: +80%; 2 g/kg: +286%, *p* < 0.01; 4 g/kg: +860%, *p* < 0.0001; Figure 6A) but reduced plasma GIP levels (1 g/kg: −40%, *p* < 0.05; 2 g/kg: −51%, *p* < 0.01; 4 g/kg: −25%, ns, vs. vehicle; Figure 6B) when compared to the vehicle control group.

### 3.4 Human Proof of Concept Study

Based on the findings in mice, a first proof of concept study was performed in a small group of healthy, human participants (*n* = 16), subdivided into a normoglycemic (*n* = 7) and a prediabetic group (*n* = 9). The description of baseline parameters is reported in Appendix S3.

**FIGURE 7:**
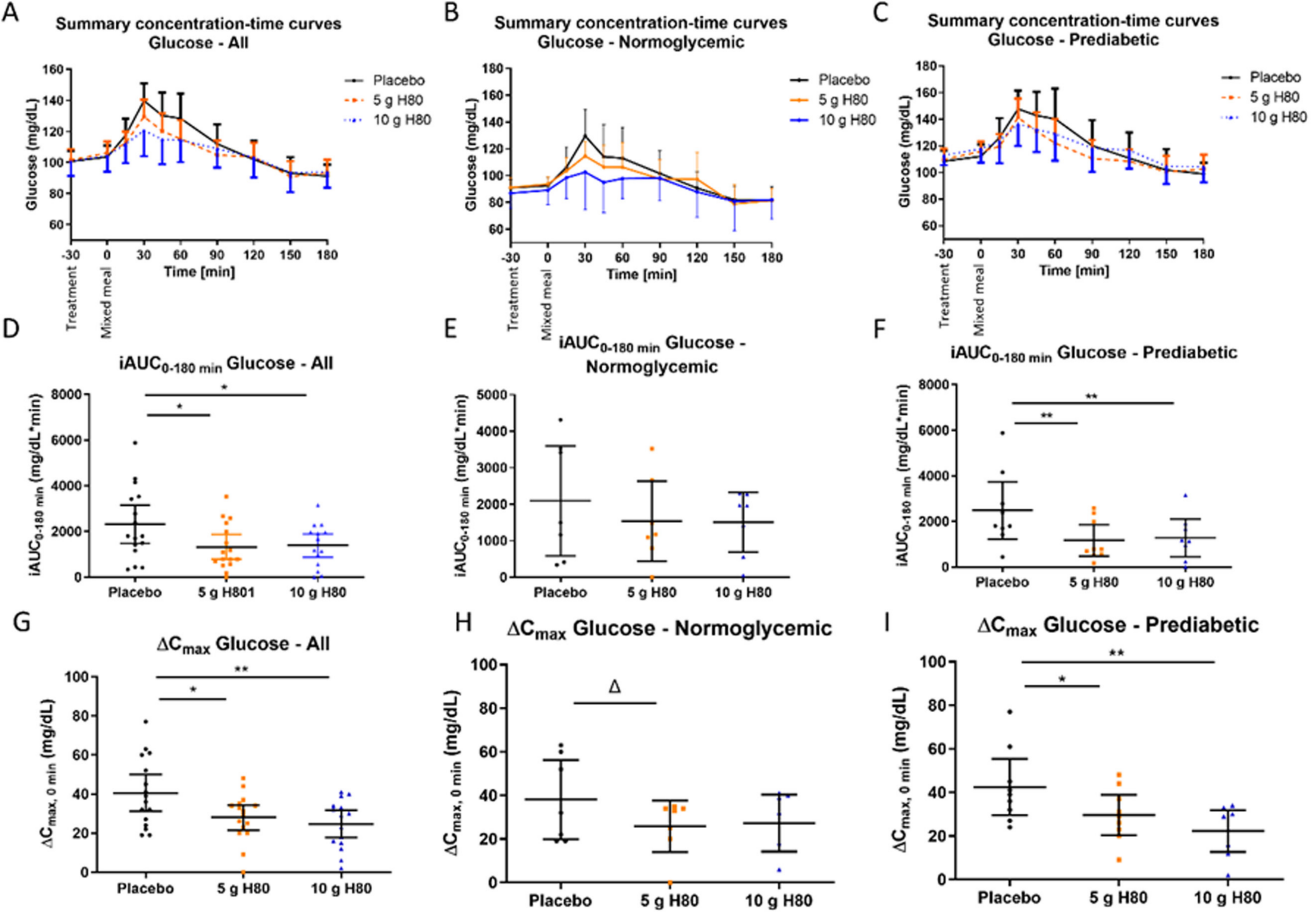
Effects of H80 on glycemia during a mixed meal test in normoglycemic and prediabetic human participants. Sixteen healthy participants were orally administered 30 min before a carbohydrate‐rich, mixed meal: Placebo, H80 at 5 g or 10 g. Panels (A, D, G) show results of the total population (*n* = 16), panels (B, E, H) of the normoglycemic subgroup (*n* = 7), panels (C, F, I) of the prediabetic subgroup (*n* = 9). (A, B, C) Summary concentration‐time curves of blood glucose over time with treatment administration at T‐30, and mixed meal intake at 0 min. (D, E, F) Incremental AUC for glucose in response to the mixed meal. (G, H, I) Maximum glucose concentration corrected for baseline. All values are means ± 95% CI. Statistical significance was evaluated by linear mixed model with multiple paired comparisons of least squares means against placebo for iAUC. Δ*p* < 0.1, *p < 0.05, **p < 0.01, ***p < 0.001.

The summary concentration‐time curves descriptively indicate that the postprandial blood glucose profile is on average decreased after intake of either dose of H80 in comparison to placebo. This is confirmed for the total study population, (Figure 7A, *n* = 16) and also for the subgroups of normoglycemic (Figure 7B, *n* = 7) and prediabetic individuals (Figure 7C, *n* = 9).

For the overall study population, the postprandial glucose response, iAUC_0–180min_, was significantly reduced (Figure 7D) after intake of 5 g H80 (−43%, treatment difference − 980 mg/dL*min (95% CI: −1737; −223), *p* = 0.0131) and 10 g H80 (−40%, −961 mg/dL*min (95% CI: −1730; −192), *p* = 0.0162) in comparison to placebo, confirming the postprandial glucose‐lowering effect of H80 observed in mice. Investigating the effects in subgroups of normoglycemic and prediabetic individuals confirmed the findings in both subgroups with a reduced postprandial glucose response after intake of H80, whereas the effects were more pronounced in the prediabetic subgroup. iAUC_0–180min_ showed a non‐significant decrease after intake of 5 g H80 (−27%, ns) and 10 g H80 (−28%, ns) in the normoglycemic group (Figure 7E). In prediabetic group a significantly reduced iAUC_0–180min_ was observed for intake of 5 g H80 (−53%, *p* = 0.0020) and 10 g H80 (−48%, *p* = 0.0028) in comparison to placebo (Figure 7F). Interestingly, no dose‐dependency was observed for the iAUC_0–180min_ between 5 g CH and 10 g H80 resulting both in a comparable postprandial reduction of glucose over time.

The maximum increase of glucose concentration (Δ*C*_max_) was determined (Figure 7G–I). In line with the above results, both 5 g and 10 g of H80 (Figure 7G, −31% with *p* = 0.0110 and −39% with *p* = 0.0017, respectively) significantly decreased Δ*C*_max_ after intake of the mixed meal in comparison to placebo for the overall study population, suggesting that H80 can reduce glucose spikes in response to a meal. No significant differences were seen between 5 g and 10 g H80. Looking at the subpopulations, H80 showed a non‐significant trend towards a decreased Δ*C*_max_ for both doses of H80 in the normoglycemic group (−32% for 5 g H80 (*p* = 0.0838), −28% for 10 g H80, ns, Figure 7H). In the prediabetic group (Figure 7I) a significantly decreased Δ*C*_max_ was observed for 5 g of H80 (−30%, *p* = 0.0170) and 10 g of H80 (−47%, *p* = 0.0013). In contrast to iAUC, data indicate a dose dependency of Δ*C*_max_ especially in the prediabetic group 5 g vs. 10 g, *p* = 0.0594.

## 4 Discussion

The control of blood glucose levels, particularly after a meal, is a critical point in normoglycemic and prediabetic individuals. Indeed, as explained in the introduction, high glucose spikes or frequent extreme amplitudes of blood glucose concentrations (a high glucose variability) can constitute a risk. Incretins, as GLP‐1 and GIP, are secreted in response to a meal and constitute targets to improve the control of postprandial blood glucose levels. One study however showed that targeting GIP alone was insufficient to lower glucose in a diabetes type 2 context, while another study identified GLP‐1 secretion and gastric emptying, among numerous other glucoregulatory factors, as the most relevant determinants of postprandial glycemia (Wu et al. 2014; Xie et al. 2023). This indicates that targeting endogenous GLP‐1 secretion in combination with gastric emptying is a valid approach to achieve glycemic control. Particularly the use of hydrolysed proteins to modulate this response is interesting as they can directly target the enteroendocrine system to enhance GLP‐1 release (Miguens‐Gomez et al. 2021). Based on these facts and the knowledge that GLP‐1, by itself, can regulate gastric emptying (Marathe et al. 2011), a specific collagen hydrolysate was selected in vitro, based on its GLP‐1 stimulating capacity with the purpose to enable glycemic control in a physiological context. The present study demonstrated that specifically developed collagen hydrolysates with a targeted bioactivity were indeed able to directly induce GLP‐1 release from murine STC‐1 cells after in vitro gastrointestinal digestion. Interestingly, the in vitro screening showed that one collagen hydrolysate, H80, was far more efficient than the others. Indeed, studies have suggested that the hydrolysis conditions applied to food proteins may lead to differences in GLP‐1 secretion, likely by generating a specific pool of bioactive peptides that trigger the GCPRs on intestinal enteroendocrine L‐cells (Miguens‐Gomez et al. 2021; Patil et al. 2024). Further mechanistic understanding of which peptides of H80 are responsible for the higher GLP‐1 secretion is an interesting subject for future research.

To validate that H80, selected as the most promising candidate in vitro, can trigger a physiologically relevant response (e.g., glucose tolerance), the product was further tested in vivo. In lean, normoglycemic mice, collagen hydrolysate H80, orally administered 45 min before an oral glucose load, reduced postprandial blood glucose excursion and increased plasma insulin as observed with the positive control sitagliptin. While the lowering of glucose by H80 at the dose of 4 g/kg was similar to that of sitagliptin, the rise of insulin was greater after sitagliptin. This suggests a different mechanism of action such as slowing of gastric emptying (Smedegaard et al. 2023; Wu et al. 2023). It was further confirmed that H80 could indeed stimulate GLP‐1 secretion into the portal vein in normoglycemic mice. When administered daily for 6 weeks in overweight, prediabetic mice, H80 decreased blood glucose levels during an oral glucose load, slowed gastric emptying and increased plasma insulin and active GLP‐1 levels. While the observed increase in ratio between insulin levels and glycemia 15 min after the glucose load points towards an incretin effect, further research will be needed to fully substantiate this finding. In this context, the investigation of the effect of H80 on insulin secretion in the absence of a test meal could provide more valuable and conclusive insights into the full set of mechanisms at play.

In both mouse models used in this study, H80 did not increase but decrease plasma active GIP levels. This effect can be expected since GLP‐1 inhibits GIP secretion (Pederson et al. 1998). In addition, the observed reduction in post‐load GIP levels could be a reflection of delayed gastric emptying, as the latter is a key determinant of GIP secretion from the proximal small intestine (Wu et al. 2013b). Our data indicate a potential for the collagen hydrolysate H80 to be used as a nutritional supplement to help control postprandial glucose levels through improved insulin secretion and delayed gastric emptying, in normoglycemic and prediabetic people, if H80 is administered before a meal.

By administering the product before the meal, it serves as a preload and is priming the body for the subsequent meal. This approach, although not generally prescribed, has already shown interesting results (Wu et al. 2016). In humans, ingestion of macronutrients (Wu et al. 2012, 2013a), like proteins (Wu et al. 2016, 2023; Ma et al. 2009), 30 min before a carbohydrate meal decreases postprandial glucose levels by 28%–50% by increasing blood GLP‐1, GIP and insulin levels and slowing gastric emptying (Sun et al. 2017; Jakubowicz et al. 2014; Wu et al. 2012; Wu et al. 2013a, 2013b; Ma et al. 2009; Wu et al. 2016; Wu et al. 2023). The timing of the preload in mice was set at 45 min before the meal because their digestion generally takes 30–60 min. Our data in both mice models (Figures 3A and 6A) illustrates that GLP‐1 secretion is significantly increased already 30 min after H80 intake, making the body ready to receive the meal. The downside of using nutrients as a preload to reduce postprandial glucose excursion is the additional energy intake it provides, potentially leading to increased body weight (Wu et al. 2012). However, the energy content of protein is not substantial when compared to the energy provided by carbohydrates or lipids. Additionally, we observed that H080 administered daily in overweight mice did not increase their body weight.

To further explore the relevance of the preclinical findings in humans, the effect of H80 on postprandial glucose was tested in a small cohort of healthy humans comprising a normoglycemic and a prediabetic subgroup. The primary objective of the study was to investigate the change in postprandial glucose iAUC between baseline and 180 min in response to a mixed meal test when either 5 g or 10 g H80 was administered 30 min before the test meal. The preload timing in humans was based on experience in the mice models and relevant literature showing that a protein preload taken 30 min before the actual meal showed the most promising results (Wu et al. 2016, 2023; Ma et al. 2009). On the other hand, our mouse data showed that GLP‐1 was increased 30 min after a protein preload. For the postprandial assessment a carbohydrate‐rich meal instead of glucose was chosen to mimic the physiological response of the gastrointestinal system to a meal including relevant elements of the incretin system. Consistent with previous studies (Wu et al. 2023), our results show that the effect on the glucose spikes is the strongest between 30 and 60 min after the meal implying that slowing down gastric emptying plays a key role in the manifestation of the glucose‐lowering effect. Despite the small sample size, the glucose lowering effects of 5 g and 10 g H80 were confirmed in the overall study population (−43% and −40%, respectively) and both normoglycemic (−27% and −28%, respectively) and prediabetic subgroups (−53% and −48%, respectively). However, significance level was only reached in the overall study population and in the prediabetic subgroup. It might be that in a somewhat derailed metabolic situation, the impact of the H80 induced glucose lowering effects is more pronounced. On the other hand, it is likely that increasing the smaller sample size in the normoglycemic group also affects the chances of obtaining a significant result.

While this study represents a first exploration of the effects of H80 in a small human cohort, a clinical study with a larger sample size is warranted to evaluate these findings and to further explore the potential of H80. Furthermore, investigating the combination of H80 with high glycemic foods may be of interest to potentially counteract a high postprandial glucose load and its negative implications. In addition, long‐term effects on glucose metabolism could be investigated to further explore its beneficial effect on metabolic health.

Here, the human study was designed to investigate glycemic effects in healthy normoglycemic and prediabetic individuals, but the question remains on how such approach could impact individuals with more advanced obesity or type 2 diabetes. A study showed that type 2 diabetes patients receiving a high concentrated protein solution 30 min before a meal for 12 weeks had reduced blood glucose levels, Hb1Ac, plasma lipid levels and inflammation markers, including CRP (Li et al. 2015). Interestingly, combining the DPP‐4 inhibitor vildagliptin with a protein preload contributed to enhance the effect of vildagliptin (Wu et al. 2016). It would be interesting to test whether H80 supplementation can modulate type 2 diabetes and its comorbidities like fatty liver, dyslipidemia, nephropathy or peripheral neuropathy, particularly since GLP‐1 based therapies have shown beneficial effects on these metabolic complications (Przezak, Bielka, and Pawlik 2021; Yaribeygi et al. 2021; Razavi et al. 2022; Mantovani et al. 2021).

H80 was selected based on its direct GLP‐1 inducing capacity in vitro. However, to achieve its glucose lowering effects, it is not excluded that more mechanisms could be at play in vivo: (i) inhibit alpha‐glucosidase (Lu et al. 2023); (ii) slow down the transportation of glucose into the small intestine, resulting in delayed glucose absorption; (iii) suppress the absorption of glucose in the small intestine (Dugardin et al. 2021) (iv) and to inhibit DPP‐IV in the blood and thereby indirectly elevate GLP‐1 levels in the circulation. However, none of the earlier reported papers on collagen hydrolysates selected for enhancing natural GLP‐1 directly. Nor has it been shown that, regarding stimulation of GLP‐1, differences exist among different hydrolysates. As H80 directly stimulates enteroendocrine cells to secrete GLP‐1, looking into potential synergistic mechanisms is an attractive approach for future research.

In addition, as soon as a nutritional approach is used, a modulation of gut microbiota should be taken into consideration as a potential contributing mechanism as well, due to the role of the gut microbiome in determining metabolic health. Indeed, protein ingestion can modulate the gut microbiota composition and activity by enhancing proteolytic microbes (Zhao et al. 2019). It would be relevant to investigate how the chronic supplementation with H80 might impact the gut microbiota composition of a high‐fat fed, overweight, prediabetic mouse, because it influences GLP‐1 action (Grasset et al. 2017; Clemmensen et al. 2013). A study in mice showed that a high‐fat, high‐sucrose diet supplemented with fish extracts highly enriched in collagen induced an improvement of glucose tolerance and enhanced beneficial gut bacteria (Axarlis et al. 2021). Collagen is enriched in hydroxyproline and hydroxylysine obtained from proline and lysine hydroxylation. For example, it was shown that poly‐lysine peptide administered orally and by intra‐duodenal injection reduced glycemia and slowed down gastric emptying through the calcium sensing receptor CaSR (Muramatsu et al. 2014) expressed by L‐cells (Acar et al. 2020; Hjorne, Modvig, and Holst 2022). Therefore, it would be interesting to investigate whether, besides the direct stimulatory effect on GLP‐1 secretion, additional and/or synergistic mechanisms could be at play to explain the physiological effects of H80. More specifically, the assessment of gastric emptying, GLP‐1, GIP, insulin and glucagon would help further elucidate the specific mechanisms of action in the human set‐up.

As mentioned before, for a protein‐based intervention collagen is an attractive target to start from as multiple mechanisms have been observed. On top of that, we have selected the most optimal conditions to further enhance the protein‐induced effect. Overall, the data indicate that the specific collagen hydrolysate, H80, has significant GLP‐1‐mediated effects on oral glucose tolerance in lean, normoglycemic and overweight, prediabetic mice. H80 also reduced the postprandial glucose response at dosages as low as 5 g in a small, human, proof of concept study. Additional research is needed to further elucidate the full potential of this specific collagen hydrolysate in supporting metabolic health.

## Author Contributions

**Estelle Grasset:** conceptualization (equal), formal analysis (equal), investigation (equal), methodology (equal), project administration (equal), resources (equal), supervision (equal), validation (equal), writing – original draft (equal). **François Briand:** conceptualization (equal), formal analysis (equal), investigation (equal), methodology (equal), project administration (equal), resources (equal), supervision (equal), validation (equal), writing – original draft (equal). **Nicolina Virgilio:** conceptualization (equal), formal analysis (equal), investigation (equal), methodology (equal), project administration (equal), resources (equal), supervision (equal), validation (equal), writing – original draft (equal). **Christiane Schön:** conceptualization (equal), formal analysis (equal), investigation (equal), methodology (equal), project administration (equal), resources (equal), validation (equal), visualization (equal). **Manfred Wilhelm:** formal analysis (equal), investigation (equal), methodology (equal), resources (equal), supervision (equal), validation (equal). **Benoit Cudennec:** conceptualization (equal), formal analysis (equal), funding acquisition (equal), investigation (equal), methodology (equal), project administration (equal), resources (equal), supervision (equal), validation (equal), writing – original draft (equal). **Rozenn Ravallec:** conceptualization (equal), formal analysis (equal), investigation (equal), methodology (equal), project administration (equal), resources (equal), supervision (equal), validation (equal). **Hairati Aboubacar:** formal analysis (equal), investigation (equal), methodology (equal), project administration (equal), resources (equal), supervision (equal), validation (equal). **Sara Vleminckx:** formal analysis (equal), investigation (equal), methodology (equal), project administration (equal), resources (equal), supervision (equal), validation (equal), writing – original draft (equal). **Janne Prawitt:** conceptualization (equal), formal analysis (equal), funding acquisition (equal), investigation (equal), methodology (equal), project administration (equal), resources (equal), supervision (equal), validation (equal), writing – original draft (equal). **Thierry Sulpice:** writing – original draft (equal). **Elien Gevaert:** conceptualization (equal), formal analysis (equal), funding acquisition (equal), investigation (equal), methodology (equal), project administration (equal), resources (equal), supervision (equal), validation (equal), writing – original draft (equal).

## Conflicts of Interest

Elien Gevaert, Sara Vleminckx, Janne Prawitt and Nicolina Virgilio are employees of Rousselot BV. Estelle Grasset, François Briand, Thierry Sulpice are employees of Physiogenex SAS, Christiane Schön is an employee of BioTeSys GmbH.

## Supporting information


Appendix S1.

## Data Availability

The data that support the findings of this study are available upon reasonable request from the corresponding author.
