# Supplementary material for: A Specific Collagen Hydrolysate Improves Postprandial Glucose Tolerance in Normoglycemic and Prediabetic Mice and in a First Proof of Concept Study in Healthy, Normoglycemic and Prediabetic Humans
Source: Food Sci Nutr. 2024 Oct 20;12(11):9607–20. doi: 10.1002/fsn3.4538 (PMC11606891; doi:10.1002/fsn3.4538)

**Supplementary Information (SI)**

**Appendix S1. Experimental design of the lean, normoglycemic mouse model.** During the complete experiment mice were fed a standard diet. After 5 days of acclimation, mice were randomized into the 4 treatment groups (n=10 mice/group) according to their body weight. Mice were administered orally with vehicle and H80 at different doses, once, 45 minutes before the oral glucose load of the oral glucose tolerance test. Plasma insulin levels were measured 45 minutes before and 15 minutes after the oral glucose load. One week after the oral glucose tolerance test, mice were administered with vehicle and H80 at different doses, once, 15 or 30 minutes before blood collection in the portal vein to assay active GLP-1 and GIP plasma levels.


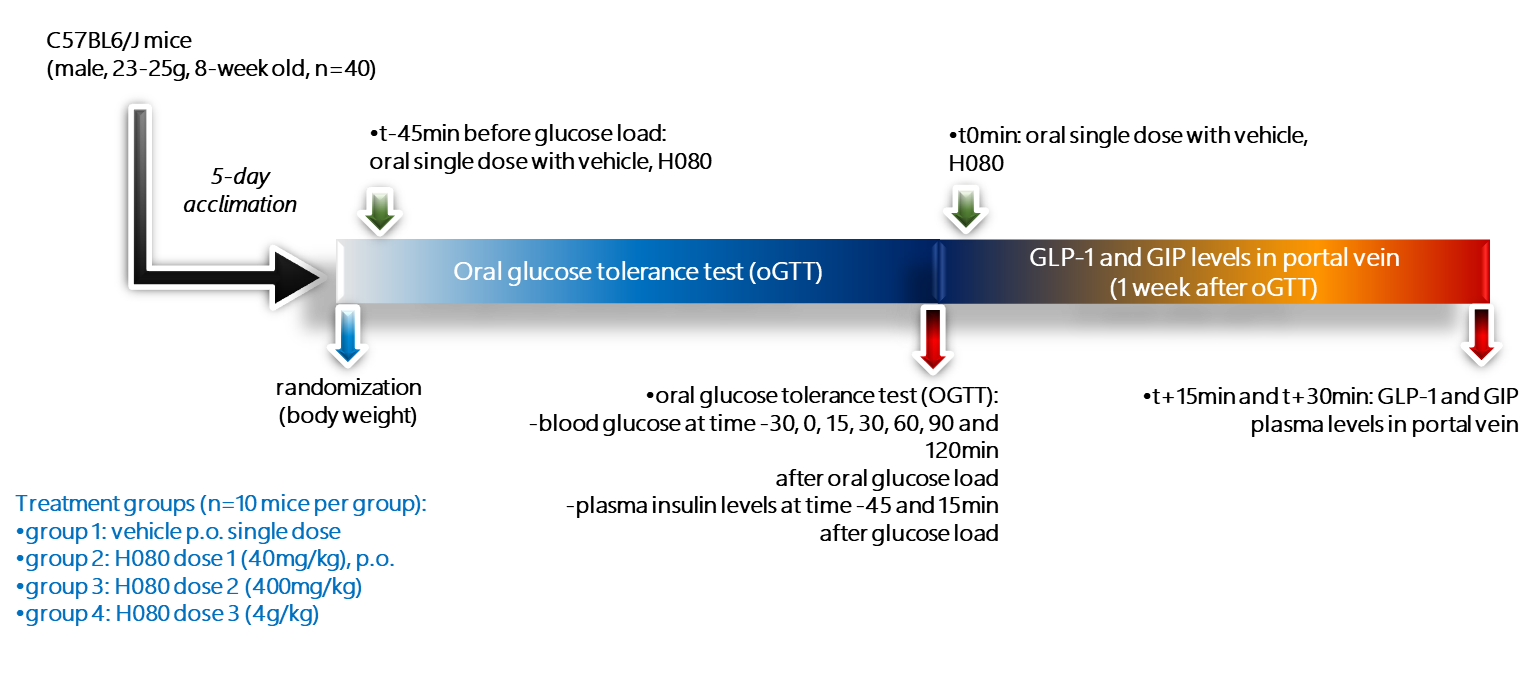


**Appendix S2: Experimental design of the diet-induced, overweight, prediabetic mouse model.** After the acclimation period, mice were fed with a 60% high-fat diet for 6 weeks. Body weight was monitored weekly. At 6 weeks of diet, mice were randomized into the 4 treatment groups (n=10 mice/group) according to their 1) HOMA-IR and 2) body weight. 10 high-fat fed mice showing extreme values (maximum and/or minimum) of HOMA-IR and body weight were excluded from the study. Mice were daily supplemented (orally) for 6 weeks with either vehicle or H80 at 3 different doses, QD (first day of treatment: day 0) while continuing the high-fat diet feeding. Body weight was monitored weekly. At day 20 of supplementation, an oral glucose tolerance test/gastric emptying assay was performed. At day 34 of supplementation, an oral glucose tolerance test was performed. At day 41 of supplementation, blood from the portal vein was collected to assay active GLP-1 and GIP plasma levels.


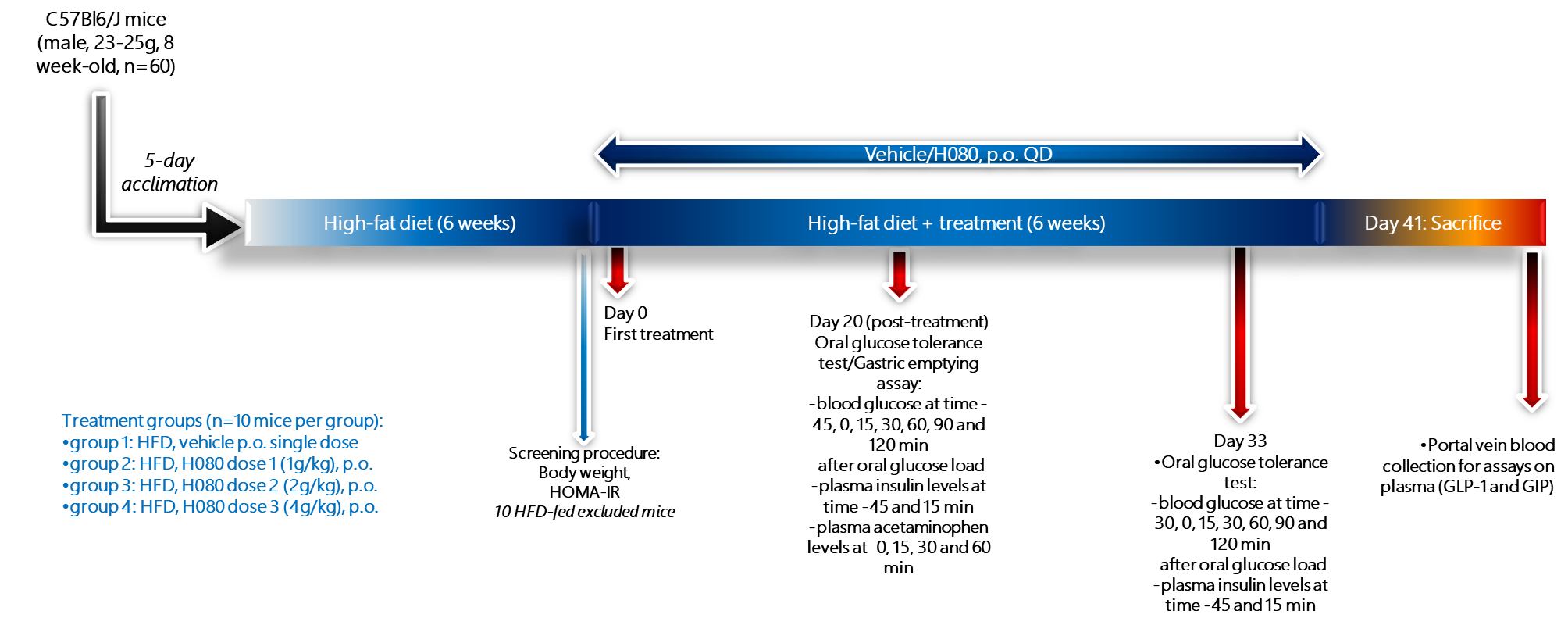


**Appendix S3: Consort flow chart**


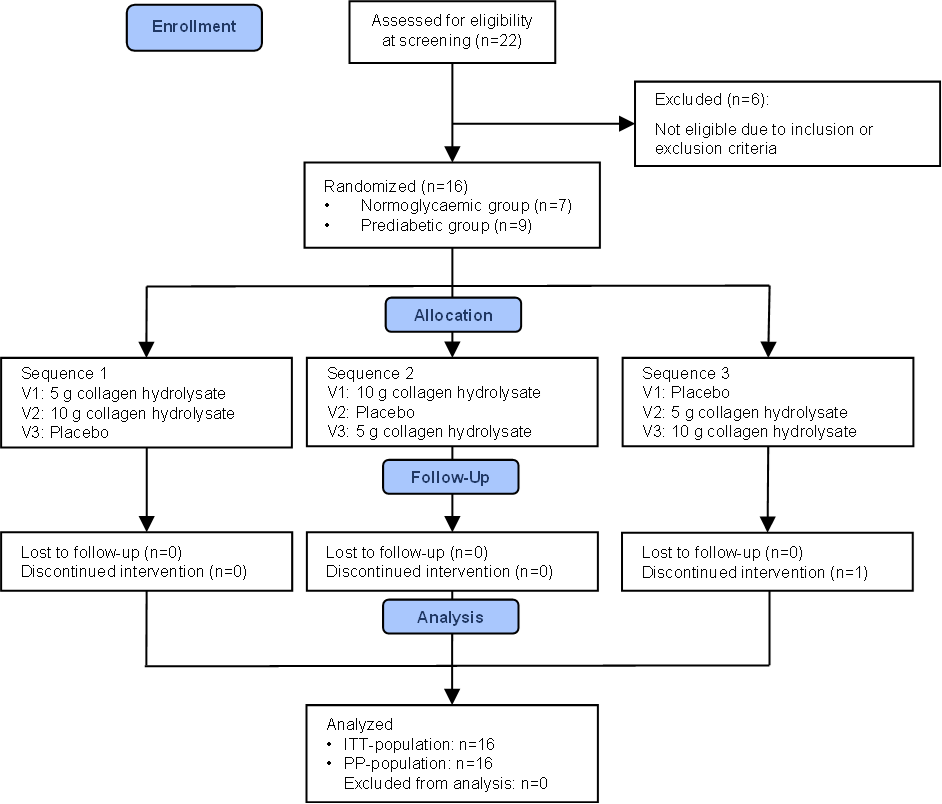


**Appendix S4 Eligibility criteria and selection of human study population**

Inclusion criteria prediabetic subjects

Male and female subjects with prediabetic HbA1c values between 5.7% and 6.4% and/or fasting glucose ≥ 100 mg/dL and ≤ 125 mg/dL (in venous plasma) (twice confirmed at two independent days if HbA1c is < 5.7%)

Inclusion criteria normoglycemic subjects

- fasting glucose < 100 mg/dL and HbA1c < 5.7%
- Age: 18-70 years
- Body mass index 19-35 kg/m^2^
- Current non-smoker
- Signed informed consent form
- No changes in food habits or physical activity 3 months prior to screening and during the study
- If applicable, stable intake of chronic medication of at least 4 weeks

Exclusion criteria

- Subjects with diagnosed Type 2 Diabetes mellitus with medical treatment
- Presence of disease or drug(s) influencing digestion (incl. recent intake of antibiotics) and absorption of nutrients
- Intake of medications known to affect glucose tolerance, e.g. diabetic medication, SGLT-2 inhibitors, GLP-1 receptor agonists, steroids, protease inhibitors or antipsychotics
- Chronic intake of substances affecting blood coagulation (e.g. acetylic acid (100 mg as standard prophylactic treatment allowed when dose is stable 1 month prior to screening), anticoagulants, diuretics, thiazides (diuretics and thiazides allowed e.g. for hypertension treatment when dose is stable 1 month prior to screening)), which in the Investigator’s opinion would impact patient safety
- Severe liver or renal disease or laboratory evidence of hepatic dysfunction (i.e. alkaline phosphatase, ALT, AST >3 x ULN)
- Known inflammatory or malignant gastrointestinal diseases (i.e. colitis ulcerosa, Morbus Crohn, celiac disease, malignant diseases e.g. colon-cancer, rectum cancer, pancreatitis)
- Clinically relevant findings as established by medical history, physical examination, clinical laboratory and/or vital signs
- Major medical or surgical event requiring hospitalization within the previous 3 months
- Intake of food supplements known to affect glucose tolerance, e.g. cinnamon capsules, conjugated linoleic acids
- Drug-, alcohol- and medication abuses
- Pregnant or breast-feeding women
- Weight loss intervention or recent body weight change >5 kg during last 3 months
- Blood donation within 4 weeks prior to Visit 1 or plan to donate blood during the study
- Anticipating any planned changes in lifestyle for the duration of the study
- Participation in another clinical intervention study within the last 4 weeks and concurrent participation in another intervention clinical study
- Subjects considered inappropriate for the study by investigators, including subjects who are unable or unwilling to show compliance with the protocol

**Table S1. Demographic and baseline characteristics of the enrolled human participants. (Data depicted as mean ± standard deviation)**

|  | **Overall** | **Normoglycemic** | **Prediabetic** |
| --- | --- | --- | --- |
| **Number of subjects** | 16 | 7 | 9 |
| **Gender (M/F)** | 5/11 | 1/6 | 4/5 |
| **Age (years)** | 57.6 ± 7.8 | 54.6 ± 8.7 | 59.9 ± 6.6 |
| **BMI (kg/m^2^)** | 26.1 ± 3.4 | 24.5 ± 3.3 | 27.4 ± 3.1 |
| **Baseline fasting glucose (venous) (mg/dL)** | 102.8 ± 12.3 | 92.3 ± 3.4 | 111.0 ± 10.0 |

**Figure S1: Body weight (A) and body weight change (B) during the high-fat diet period (6 weeks) before starting supplementation in overweight, prediabetic mice.**


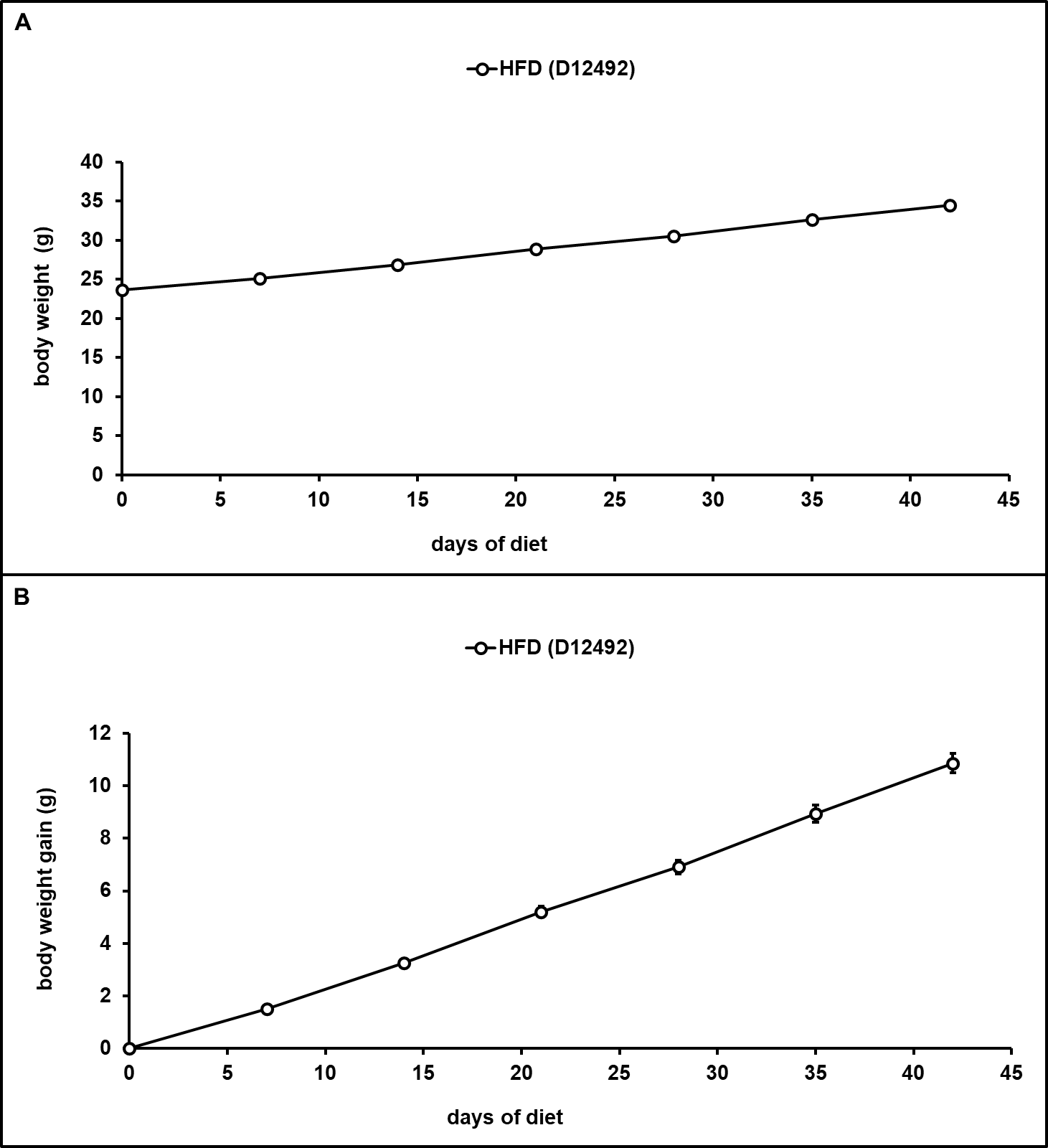


**Figure S2: Body weight (A) and HOMA-IR (B) of high-fat fed, overweight, prediabetic mice at randomization/supplementation start.**


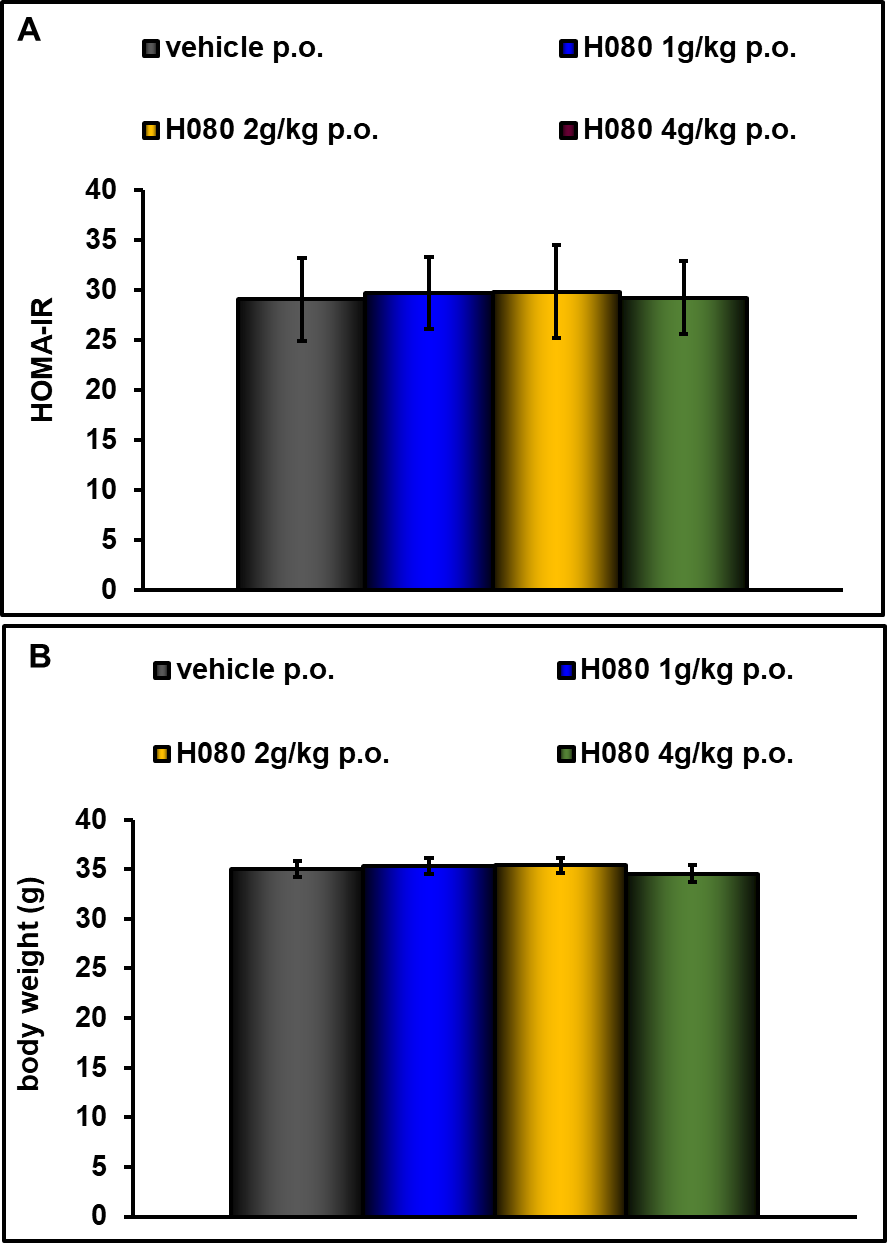


**Figure S3: Body weight (A) and body weight change (B) during the 6-week supplementation period in overweight, prediabetic mice.** Data are mean ± SEM, n=6-10 per group. Statistical significance was evaluated by two-way ANOVA (followed by posthoc Bonferroni’s multiple-comparison tests) *p<0.05, **p<0.01, ***p<0.001, ****p<0.0001


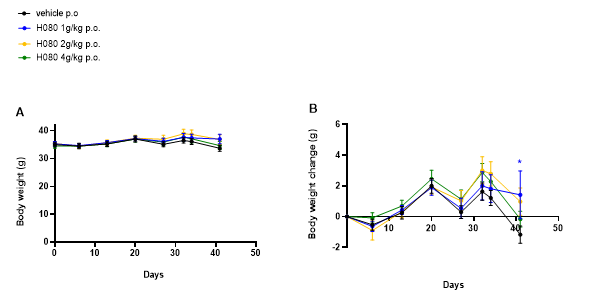

Supplement: Supplementary file 1 — Appendix S1. [file FSN3-12-9607-s001.docx]
